# Supplementary material for: Mitochondrial network configuration influences sarcomere and myosin filament structure in striated muscles
Source: Nat Commun. 2022 Oct 13;13:6058. doi: 10.1038/s41467-022-33678-y (PMC9561657; doi:10.1038/s41467-022-33678-y)
Supplement: Supplementary file 1 — Supplementary Info [file 41467_2022_33678_MOESM1_ESM.pdf]

SUPPLEMENTARY INFORMATION

Mitochondrial Network Configuration Influences Sarcomere and Myosin Filament Structure in Striated Muscles

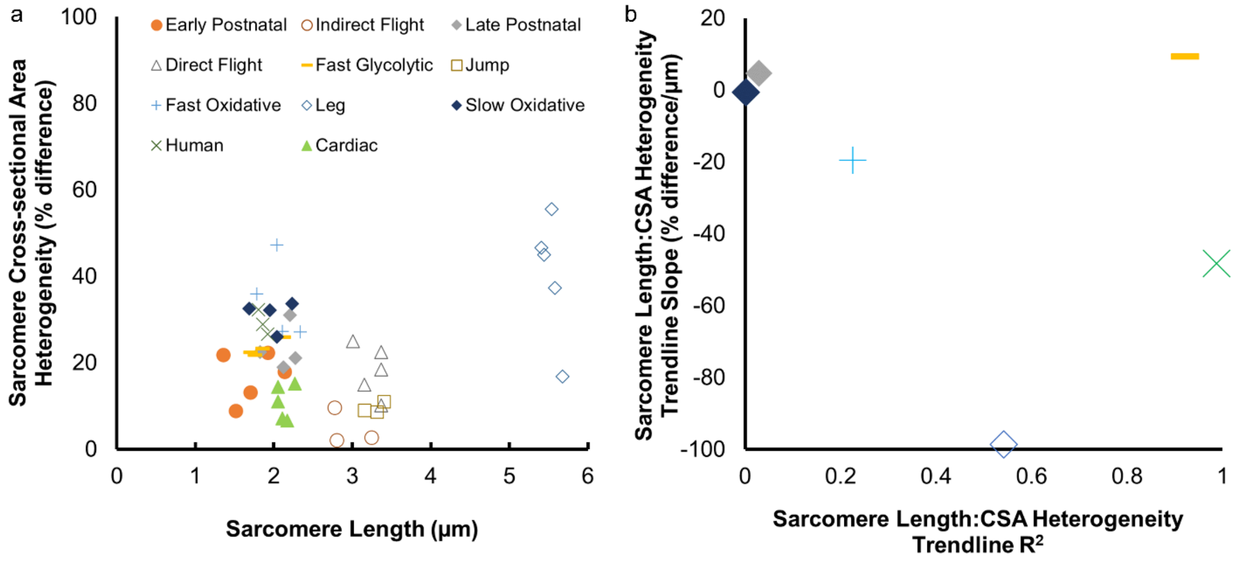

Supplementary Figure 1: Relationship between Sarcomere Length and Intrasarcomere Cross-sectional Area Heterogeneity. a) Sarcomere lengths and intrasarcomere CSA heterogeneity values corresponding to all points in Figure 2l,m. b) Slope and R^2 values for linear trendlines of the relationship between sarcomere length and intrasarcomere CSA heterogeneity for the six cell types with greater than 20% CSA heterogeneity.

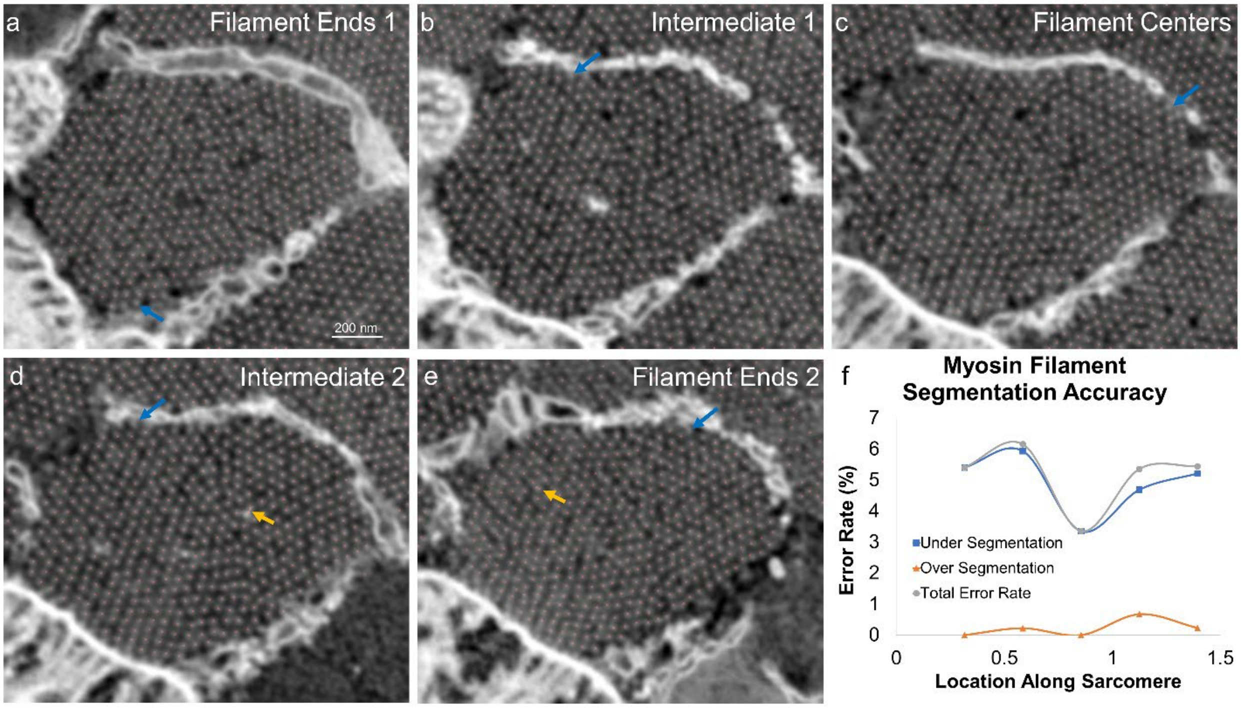

Supplementary Figure 2: Assessment of Myosin Filament Segmentation Accuracy. a-e) Raw 2D images (greyscale) overlaid with skeletons of segmented myosin filaments (red) at five locations along a single mouse late postnatal muscle sarcomere. Blue arrows highlight missed (under) segmentations. Orange arrows highlight over segmentations. f) Quantitative assessment of under, over, and total segmentation errors at five points along the sarcomere length.

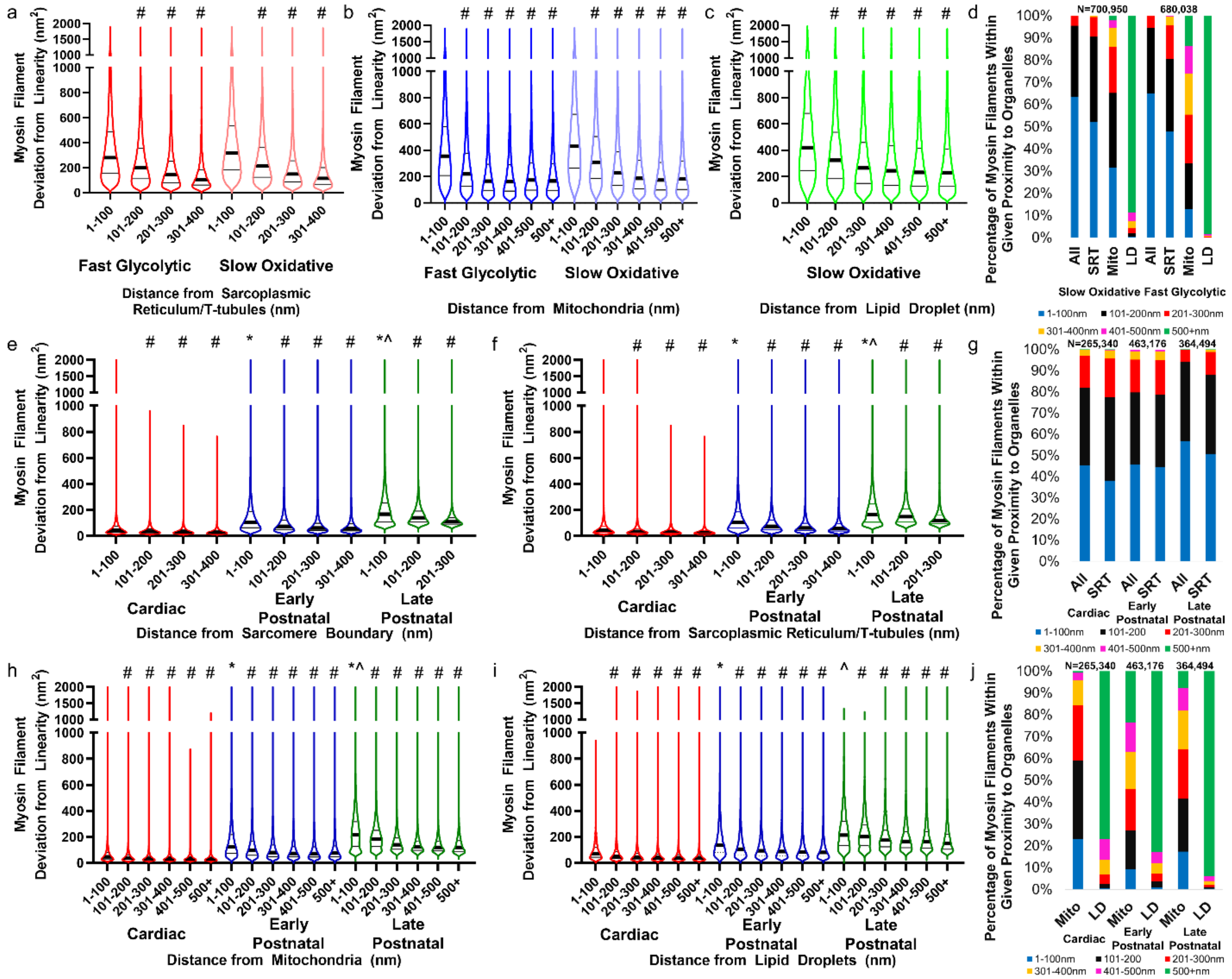

Supplementary Figure 3: Myosin filament curvature and organelle proximity in mouse muscles. a-c) Myosin filament deviation from linearity as a function of distance from sarcoplasmic reticulum/t-tubules (a), mitochondria (b), and lipid droplets (c) for Slow Oxidative and Fast Glycolytic fibers. d) Percentage of myosin filaments within 100 nm distances from sarcomere boundary organelles in Slow Oxidative and Fast Glycolytic muscles. e,f,h,i) Myosin filament deviation from linearity as a function of distance from the sarcomere boundary (e), sarcoplasmic reticulum/t-tubules (f), mitochondria (h), and lipid droplets (i) for Cardiac, Early Postnatal, and Late Postnatal fibers. g,j) Percentage of myosin filaments within 100 nm distances from sarcomere boundary organelles in Cardiac, Early Postnatal, and Late Postnatal muscles. #significantly different from 1-100 nm. *significantly different from Cardiac. ^significantly different from Early Postnatal. Two-sided, one-way ANOVA with a Tukey's HSD post hoc test.

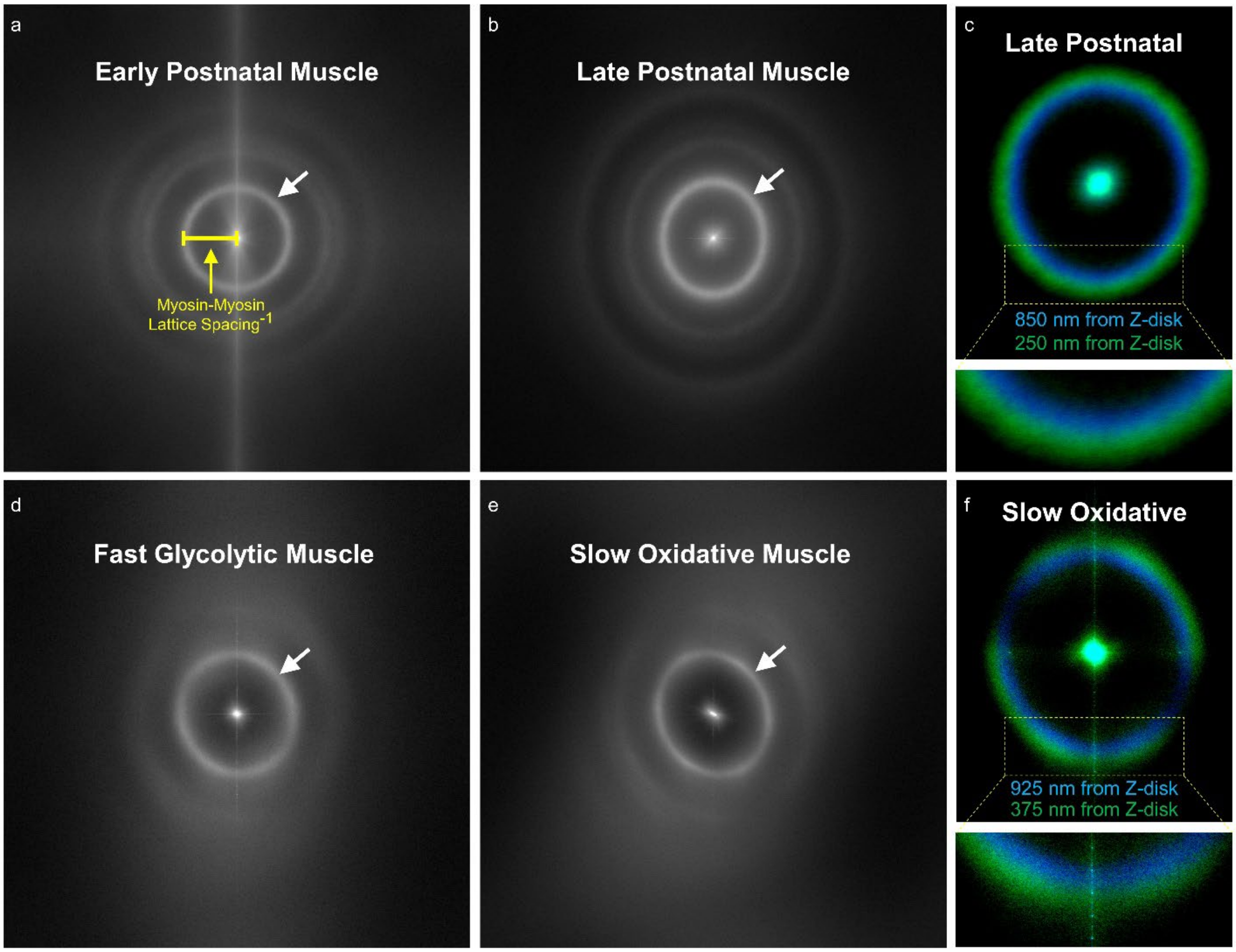

Supplementary Figure 4: 2D FFT Analyses Reveal Intrasarcomere Lattice Spacing Heterogeneity. a,b,d,e) Average of all 2D fast fourier transform (FFT) power spectrums of myosin filament center cross-section images for Early Postnatal (a), Late Postnatal (b), Fast Glycolytic (d) and Slow Oxidative (e) muscles. c,f) Average 2D FFT power spectrum of Late Postnatal (c) and Slow Oxidative (f) myosin filament centers (blue) and ends (green).

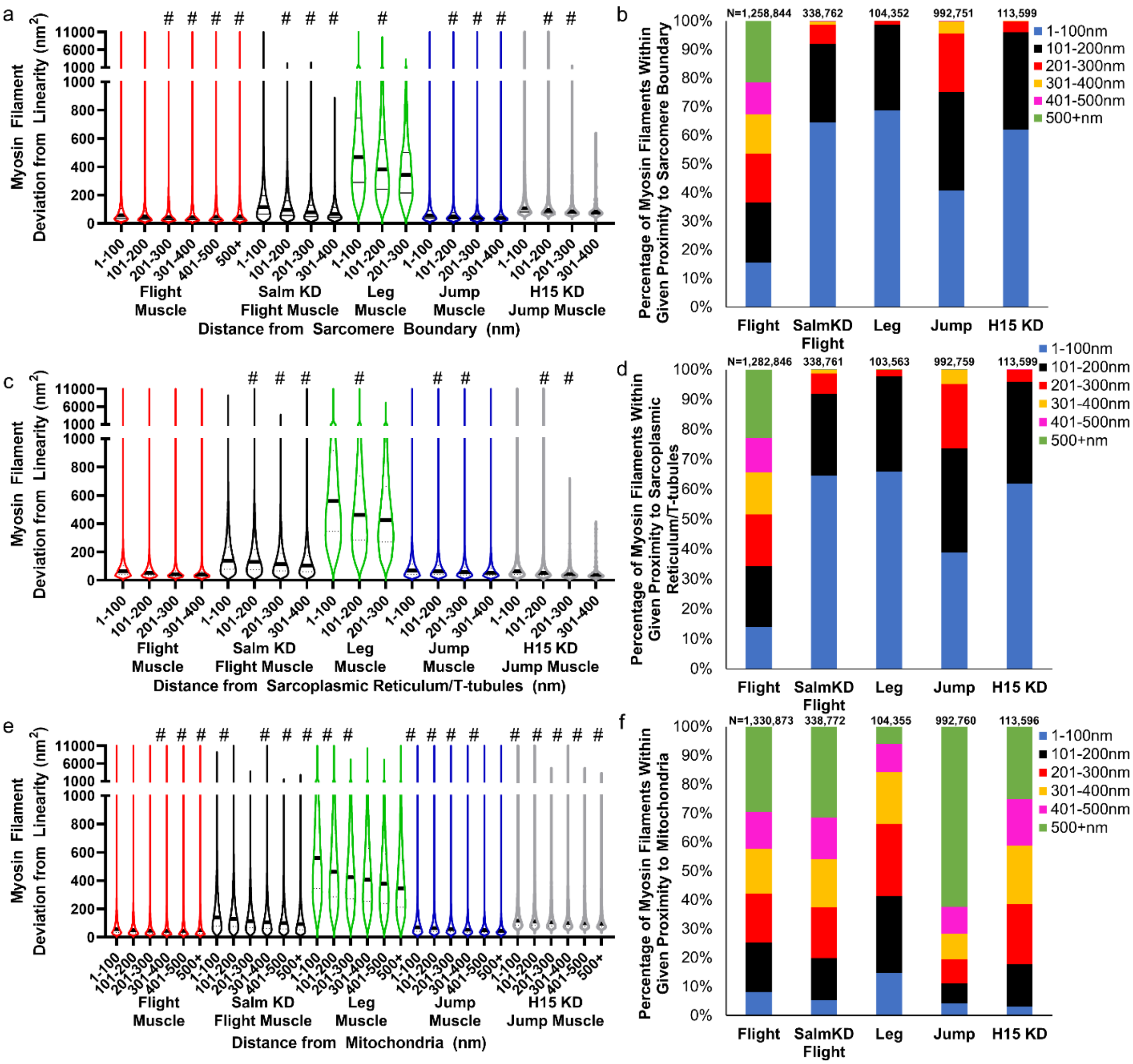

Supplementary Figure 5: Myosin filament curvature and organelle proximity in *Drosophila* muscles. a,c,e) Myosin filament deviation from linearity as a function of distance from sarcomere boundary (a), sarcoplasmic reticulum/t-tubules (c), and mitochondria (e) for Leg, Jump, H15 KD Jump, Flight, and Salm KD Flight muscles. b,d,f) Percentage of myosin filaments within 100 nm distances from sarcomere boundary organelles (b), sarcoplasmic reticulum/t-tubules (d), and mitochondria (f) in Leg, Jump, H15 KD Jump, Flight, and Salm KD Flight muscles. #significantly different from 1-100 nm. Two-sided, one-way ANOVA with a Tukey's HSD post hoc test.

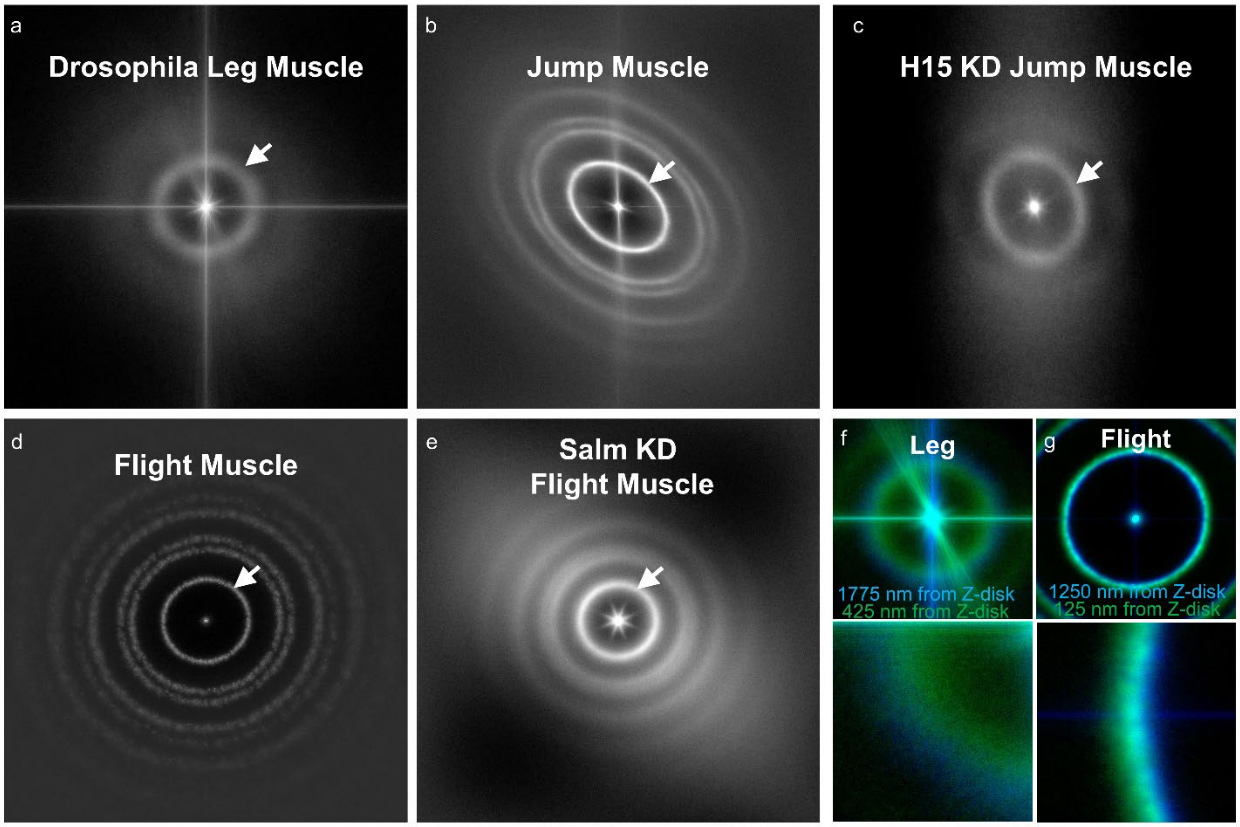

Supplementary Figure 6: 2D FFT Analyses of Myosin Lattice Spacing in *Drosophila* muscles. a-e) Average of all 2D fast fourier transform (FFT) power spectrums of myosin filament center cross-section images for Drosophila Leg (a), Jump (b), *H15* KD Jump (d), and *salm* KD Flight (e) muscles. f,g) Average 2D FFT power spectrum of Leg (f) and Flight (g) muscle myosin filament centers (blue) and ends (green).

IMAGEJ MACROS USED FOR IMAGE ANALYSIS

FFT of Image Stacks

```
//Credit: Wayne Rasband
setBatchMode(true);
   stack = getImageID;
   n = nSlices;
   for (i=1; i<=n; i++) {
      selectImage(stack);
      setSlice(i);
      run("FFT");
   }
   run("Images to Stack", "name=[FFT Stack] title=FFT");
   setBatchMode(false);
```

FFT Radial Intensity Analyses

```
setBatchMode(true);
    stack = getImageID;
  path="E:/Neighbors/IFM2/Testing/"
    for (i=1; i<=nSlices; i++) {
       showProgress(i, nSlices);
       selectImage(stack);
       setSlice(i);
       run("Duplicate...", "title=150to160intensity");
       run("Intensity Measurements 2D/3D", "input=150to160intensity labels=DistanceFromFFTCenter.tif
mean neighborsmean");
              saveAs("Results", path + "150to160intensity" + i + ".csv");
        close;
        close("150to160intensity" + i + ".csv");
    }
    setBatchMode(false);
```

Results Table Calculations

```
selectWindow("Results");
TestContent=getInfo("window.contents");
for(i=0; i<nResults; i++) {
    I3 = getResult("I3", i);
    Vol = getResult("Vol. (pixels³)", i);
    setResult("I3perVol", i, I3/Vol);
}
selectWindow("Results");
```
