# Supplementary material for: Mitochondrial network configuration influences sarcomere and myosin filament structure in striated muscles
Source: Nat Commun. 2022 Oct 13;13:6058. doi: 10.1038/s41467-022-33678-y (PMC9561657; doi:10.1038/s41467-022-33678-y)
Supplement: Supplementary file 3 — Description of Additional Supplementary Files [file 41467_2022_33678_MOESM3_ESM.pdf]

Description of Additional Supplementary Files

File Name: Supplementary Movie 1
Description: Cross-sectional view of three serial sarcomeres from two parallel myofibrillar segments from a fast twitch glycolytic adult mouse gastrocnemius muscle visualized by focused ion beam scanning electron microscopy with 6 nm isotropic pixel size.

File Name: Supplementary Movie 2
Description: 3D rendering and rotation of the adult gastrocnemius muscle sarcomeres (translucent yellow and cyan), Z-disks (red), mitochondria (magenta), sarcoplasmic reticulum (g reen), and t-tubules (blue) from the raw data shown in Supplementary Movie 1.

File Name: Supplementary Movie 3
Description: 3D rendering and rotation of sarcomeres (translucent yellow and cyan), Z-disks (red), and mitochondria (magenta) from *Drosophila* indirect flight muscle.

File Name: Supplementary Movie 4
Description: 3D rendering and rotation of the Z-disk (light green and various colors) and sarcomere (magenta) sheets that comprise the *Drosophila* jump muscle.

File Name: Supplementary Movie 5
Description: 3D rendering and rotation of sarcomeres (translucent yellow and cyan), Z-disks (red), and mitochondria (magenta) from Human vastus lateralis muscle.

File Name: Supplementary Movie 6
Description: 3D rendering and fly through of the mitochondrial networks in *Drosophila* indirect flight (green), direct flight (blue), jump (yellow), and leg (magenta) muscles.

File Name: Supplementary Movie 7
Description: 3D rendering and fly through of the grid-like mitochondrial network of a slow-twitch oxidative mouse gastrocnemius muscle. Raw FIB-SEM data (greyscale) is pulled back to reveal the mitochondrial network segmented into Z-disk adjacent (light blue) and non-Z-disk adjacent (red) mitochondria.

File Name: Supplementary Movie 8
Description: 3D rendering and fly through of the parallel mitochondrial network of a mouse cardiac muscle. The mitochondrial network is segmented into Z-disk adjacent (light blue) and non-Z-disk adjacent (red) mitochondria.

File Name: Supplementary Movie 9
Description: 3D rendering and fly through of all the myosin filaments (various colors) from a mouse late postnatal soleus muscle FIB-SEM dataset.

File Name: Supplementary Movie 10
Description: Overlay of segmented myosin filaments on raw FIB-SEM data from a mouse late postnatal soleus muscle.

File Name: Supplementary Movie 11
Description: Overlay of segmented myosin filament skeletons on the raw FIB-SEM data from a single sarcomere from a late postnatal soleus muscle.

File Name: Supplementary Movie 12
Description: 3D rendering and fly through of all the myosin filaments from a mouse late postnatal soleus muscle FIB-SEM dataset. Filaments are colored according to their deviation from linearity where more linear filaments are dark blue and more curved filaments are yellow/white.

File Name: Supplementary Movie 13
Description: 3D rendering and rotation of the myosin filaments within a single mouse cardiac sarcomere. Adjacent mitochondria (red), sarcotubular network (green), and a lipid droplet (cyan) are also shown.

File Name: Supplementary Movie 14
Description: 3D rendering and fly through of the myosin filaments (various colors) within a single mouse slow-twitch oxidative muscle sarcomere.

File Name: Supplementary Movie 15
Description: Cross-sectional view of the segmented myosin filaments (white) along the length of a single sarcomere and the corresponding 2D fast fourier transform power spectrum from mouse early postnatal, late postnatal, slow-twitch oxidative, and fast-twitch glycolytic muscles.

File Name: Supplementary Movie 16
Description: 3D rendering and 360° rotations of an adult mouse vastus lateralis muscle mitochondrial network showing acutely swollen (green) and normal (magenta) mitochondrial regions.
