# Supplementary material for: Mitochondrial network configuration influences sarcomere and myosin filament structure in striated muscles
Source: Nat Commun. 2022 Oct 13;13:6058. doi: 10.1038/s41467-022-33678-y (PMC9561657; doi:10.1038/s41467-022-33678-y)
Supplement: Supplementary file 20 — Reporting Summary [file 41467_2022_33678_MOESM20_ESM.pdf]

nature portfolio

Corresponding author(s): Brian Glancy

Last updated by author(s): Sep 21, 2022

Reporting Summary

Nature Portfolio wishes to improve the reproducibility of the work that we publish. This form provides structure for consistency and transparency in reporting. For further information on Nature Portfolio policies, see our Editorial Policies and the Editorial Policy Checklist.

Statistics

For all statistical analyses, confirm that the following items are present in the figure legend, table legend, main text, or Methods section.

n/a	Confirmed	
☐	☒	The exact sample size (*n*) for each experimental group/condition, given as a discrete number and unit of measurement
☐	☒	A statement on whether measurements were taken from distinct samples or whether the same sample was measured repeatedly
☐	☒	The statistical test(s) used AND whether they are one- or two-sided<br>*Only common tests should be described solely by name; describe more complex techniques in the Methods section.*
☐	☒	A description of all covariates tested
☐	☒	A description of any assumptions or corrections, such as tests of normality and adjustment for multiple comparisons
☐	☒	A full description of the statistical parameters including central tendency (e.g. means) or other basic estimates (e.g. regression coefficient) AND variation (e.g. standard deviation) or associated estimates of uncertainty (e.g. confidence intervals)
☐	☒	For null hypothesis testing, the test statistic (e.g. *F*, *t*, *r*) with confidence intervals, effect sizes, degrees of freedom and *P* value noted<br>*Give P values as exact values whenever suitable.*
☒	☐	For Bayesian analysis, information on the choice of priors and Markov chain Monte Carlo settings
☐	☒	For hierarchical and complex designs, identification of the appropriate level for tests and full reporting of outcomes
☒	☐	Estimates of effect sizes (e.g. Cohen's *d*, Pearson's *r*), indicating how they were calculated

Our web collection on statistics for biologists contains articles on many of the points above.

Software and code

Policy information about availability of computer code

Data collection	ZEISS Crossbeam 540 with ZEISS Atlas 5 software
Data analysis	ImageJ v1.53p, Ilastik 1.3.3post2, Imaris 9.5.0, Excel 2016, Prism 9.0.0, SPSS 28.0.0.0, Thermo Scientific Avizo 2020.3

For manuscripts utilizing custom algorithms or software that are central to the research but not yet described in published literature, software must be made available to editors and reviewers. We strongly encourage code deposition in a community repository (e.g. GitHub). See the Nature Portfolio guidelines for submitting code & software for further information.

Data

Policy information about availability of data

All manuscripts must include a data availability statement. This statement should provide the following information, where applicable:

- Accession codes, unique identifiers, or web links for publicly available datasets
- A description of any restrictions on data availability
- For clinical datasets or third party data, please ensure that the statement adheres to our policy

The raw FIB-SEM datasets generated and/or analysed during the current study are available at (https://doi.org/10.5281/zenodo.5796264).

Human research participants

Policy information about studies involving human research participants and Sex and Gender in Research.

Reporting on sex and gender	N/A
Population characteristics	N/A
Recruitment	N/A
Ethics oversight	N/A

Note that full information on the approval of the study protocol must also be provided in the manuscript.

Field-specific reporting

Please select the one below that is the best fit for your research. If you are not sure, read the appropriate sections before making your selection.

☒ Life sciences ☐ Behavioural & social sciences ☐ Ecological, evolutionary & environmental sciences

For a reference copy of the document with all sections, see nature.com/documents/nr-reporting-summary-flat.pdf

Life sciences study design

All studies must disclose on these points even when the disclosure is negative.

Sample size	Sample sizes were chosen based on previous studies using similar measurements in Bleck et al. Nat Comms, 2018 and Willingham et al. Nat Comms, 2020.
Data exclusions	Myosin filaments segmented to be less than 1 µm and/or overlapping with other organelles were not used for analyses since these are clear indicators of segmentation errors.
Replication	All studies were successfully repeated in at least two biological replicates
Randomization	Not applicable due to the lack of perturbations to randomize
Blinding	No blinding was performed due to the clear phenotype differences among muscle types.

Reporting for specific materials, systems and methods

We require information from authors about some types of materials, experimental systems and methods used in many studies. Here, indicate whether each material, system or method listed is relevant to your study. If you are not sure if a list item applies to your research, read the appropriate section before selecting a response.

Materials & experimental systems

n/a	Involved in the study	
☒	☐	Antibodies
☒	☐	Eukaryotic cell lines
☒	☐	Palaeontology and archaeology
☐	☒	Animals and other organisms
☒	☐	Clinical data
☒	☐	Dual use research of concern

Methods

n/a	Involved in the study	
☒	☐	ChIP-seq
☒	☐	Flow cytometry
☒	☐	MRI-based neuroimaging

Animals and other research organisms

Policy information about studies involving animals; ARRIVE guidelines recommended for reporting animal research, and Sex and Gender in Research

Laboratory animals	6-8 week old C57BL6/N mice were purchased from Taconic Biosciences (Rensselaer, NY) and fed ad libitum on a 12-hour light, 12-hour dark cycle at 20-26° C and 22-70% humidity. Breeding pairs were setup, and progeny were randomly selected for each experimental group. Early Postnatal mice were from postnatal day 1 (P1) and Late Postnatal mice were from P14. Adult mice were

	2-4 months of age. Animals were given free access to food and water and pups were weaned at P21.<br>W1118 (BS# 3605) flies from the Bloomington Drosophila Stock Center were crossed on yeast corn medium (Bloomington Recipe) at 22° C. Mef2- Gal4 (III) (BS# 27390) was used to drive muscle specific gene knockdown. RNAi lines for knockdown of salm (UAS-salm RNAi, V101052) and H15 (UAS-H15 RNAi, V28415) were purchased from the Vienna Drosophila Resource Center. Muscles from 2-3 days old flies were dissected.
Wild animals	No wild animals were used in this study
Reporting on sex	Due to difficulty using anogenital distance to reliably determine gender in P1 pups, we did not group mice depending on sex, but randomly used both male and female mice at all time points.
Field-collected samples	No field collected samples were used in this study
Ethics oversight	All procedures were approved by the National Heart, Lung, and Blood Institute Animal Care and Use Committee and performed in accordance with the guidelines described in the Animal Care and Welfare Act (7 USC 2142 § 13).

Note that full information on the approval of the study protocol must also be provided in the manuscript.
